# Information pharmacists assist in the construction of GLP-1RA prescription review rules in a tertiary hospital in China

**DOI:** 10.1186/s12913-025-13377-2

**Published:** 2025-10-01

**Authors:** Li Zhou, Wenjing Duanmu, Feilong Tan, Xi Gu, Hongyi Che, Wenjie Yin

**Affiliations:** Department of Pharmacy, The Affiliated Yan’an Hospital of Kunming Medical University, 245 Renmin East Road, Kunming, Panlong District, Yunnan Province China

**Keywords:** Glucagon-like peptide-1 receptor agonist, Information pharmacists, Prescription review rules, Adverse drug events, Rational drug use

## Abstract

**Background:**

Glucagon-like peptide-1 receptor agonists (GLP-1RAs) are increasingly used for type 2 diabetes mellitus (T2DM) due to their multifaceted benefits, including glycemic control and cardiovascular protection. However, variations in prescribing practices and potential adverse drug events (ADEs) necessitate standardized prescription review protocols to ensure medication safety and efficacy.

**Objective:**

This study aimed to develop and implement GLP-1RA prescription review rules through a multidisciplinary information pharmacist team, evaluate their impact on prescription rationality, and identify ADE risk signals using real-world data, thereby promoting rational clinical medication use and ensuring patient safety.

**Methods:**

A multidisciplinary information pharmacist team was established at a tertiary hospital to develop GLP-1RA prescription review protocols. China-approved GLP-1RA formulations and their clinical parameters were systematically reviewed to establish standardized prescribing criteria, including indications, dosing, and safety considerations. Adverse drug events were analyzed using FDA Adverse Event Reporting System data (2018–2023) to identify risk patterns. A dual review system integrating prospective prescription screening and retrospective evaluation was implemented. The intervention’s efficacy was evaluated by comparing prescription approval rates pre- (2022) and post-implementation (2023).

**Results:**

The GLP-1RA audit rules represented by liraglutide and semaglutide were successfully created, as well as their potential adverse event signals were successfully obtained. The process of prescription review and medication monitoring enabled them to be put into clinical practice. After the rules were put in place in 2023, the pass rates of GLP-1RA prescriptions significantly improved, and the rationalization of these prescriptions was also notably enhanced compared to the same period in 2022 (*p* < 0.001).

**Conclusions:**

Standardized GLP-1RA review rules enhanced prescription rationality and ADE risk awareness, demonstrating the value of information pharmacists in optimizing clinical decision-making. This model is scalable for other high-risk medications, promoting safer drug use and pharmacist-led innovation in healthcare.

## Contributions to the literature

1. Standardizing the prescription audit mode of GLP-1RA is very important to promote the rational use of GLP-1RA as well as to guarantee the safety of patients'medication.
2. The creation and application of GLP-1RA audit rules by hospital information pharmacists is one of the innovative pharmacy service models by pharmacists, which fully reflects the value of pharmacists'ability.
3. This is the first practical project since the formation of the information pharmacist team, which is innovative, practical and popularizable.

## Introduction

The prevalence of diabetes and prediabetes in China has been steadily increasing, rising from less than 1% in 1980 to 12.4% in recent years [1]. Notably, type 2 diabetes mellitus (T2DM) accounts for over 90% of all diabetes cases [2–4]. During the treatment of T2DM, the control of glycated hemoglobin levels is often unsatisfactory, with less than 50% of patients meeting the target, leading to a range of issues including high prevalence, poor treatment outcomes, low patient adherence, and increased economic burden [5, 6]. Therefore, this type of disease has become a health issue of great concern to the entire society.

Glucagon-like peptide-1 receptor agonist (GLP-1RA) belongs to the enteroglucagon class of drugs [7, 8]. As a new class of glucose-lowering drugs, its application in the field of T2DM treatment has become more and more widespread in recent years with the continuous improvement of clinical evidence [9–11]. Compared with traditional hypoglycemic agents, GLP-1RA has benefits such as antihypertensive, lipid regulation, weight loss, as well as cardiovascular and renal protection, in addition to glucose-related hypoglycemic effects and protection and repair of pancreatic islet β-cells [12, 13], making it the most attractive drug for the treatment of T2DM. Currently, nine GLP-1RAs have been approved for clinical use in China, with semaglutide injection and liraglutide injection being the most frequently prescribed in our institution. To optimize T2DM management, our hospital’s Pharmacy Administration and Therapeutics Committee periodically evaluates and updates the formulary based on clinical needs and patient profiles. This dynamic selection process expands therapeutic options while maximizing patient benefits through personalized treatment strategies. Since the pharmacological properties and clinical application of GLP-1RA need to be further clarified, we have to pay attention to its adverse drug reactions, drug interactions, and use in special populations in the process of using it [14–16]. For this reason, the information pharmacists of our hospital’s pharmacy department actively create audit rules for GLP-1RA drugs, effectively control the risk of GLP-1RA adverse drug events (ADEs), proactively participate in medication education and medication guidance, and standardize doctors’ prescribing behaviors. Through these multidimensional interventions, we aim to enhance prescription appropriateness of GLP-1RA therapies, promote medication safety and rational clinical use, and demonstrate the critical role of pharmacists in diabetes management.

## Methods

### Introduction of information pharmacist team

The Department of Pharmacy at The Affiliated Yan’an Hospital of Kunming Medical University has established a team of information pharmacists with five members, including one deputy chief pharmacist, three pharmacists-in-charge and one junior pharmacist. All members of the team have participated in and passed the national “Hospital Information Pharmacist Training Course”, mastered the basic knowledge of pharmacy information, and possessed the basic theoretical and practical skills of pharmaceutical information processing. All members are able to process, improve and apply hospital pharmacy information by using modern information technology with pharmacy information service as the core.

### GLP-1RA information overview

The information pharmacist collected and compiled data on all GLP-1RAs approved in China for T2DM treatment as of December 2023, and documented the basic information on their dosage forms, specifications, health insurance attributes, prescription restrictions, and whether they are national essential drugs, national negotiated drugs, and other basic information on their clinical applications.

### Establishment of GLP-1RA audit rules

**Fig. 1:**
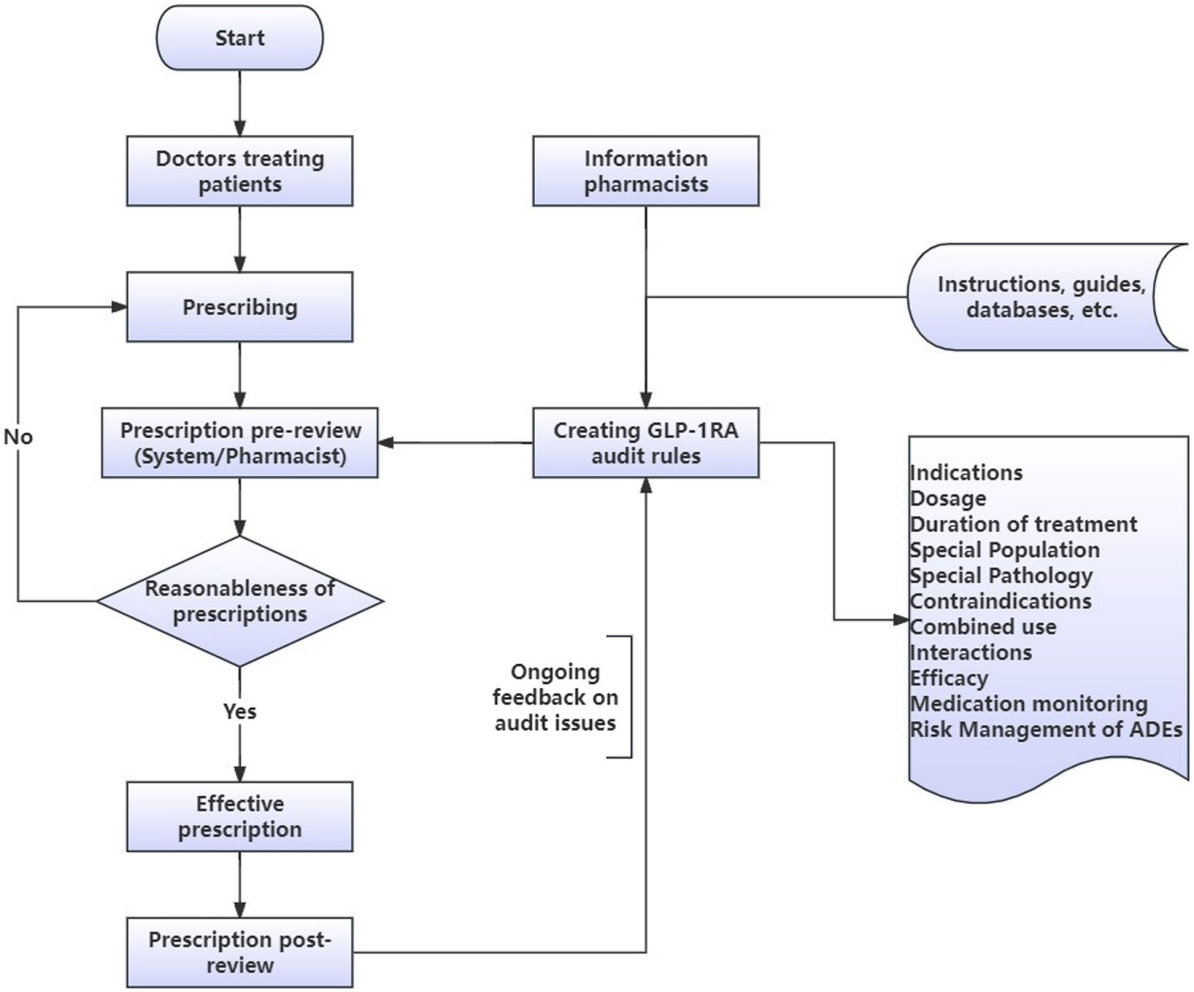
Flowchart of the information pharmacist’s efforts to help standardize GLP-1RA prescription review

As shown in Fig. 1, the information pharmacist developed standardized GLP-1RA audit rules based on drug specifications, clinical guidelines, and hospital practice. These rules encompassed indications, dosage, treatment duration, special populations, drug interactions, and ADE risk alerts. Additionally, the information pharmacist continuously monitored both prospective pre-review (conducted after prescription issuance but prior to pricing, billing, and dispensing) and prescription post-review (performed post-payment), and subsequently updated the audit rules based on identified issues. Both processes were implemented in strict accordance with China’s National Health Commission “Notice on the Issuance of Prescription Review Standards for Medical Institutions“ [17].

### Risk management of ADEs in GLP-1RAs

The information pharmacist conducted signal mining and analysis of ADE reports of GLP-1RA in the FDA Adverse Event Reporting System (FAERS) database by the Reporting Odds Ratio (ROR) and Proportional Reporting Ratio (PRR) methods [18–20], and evaluated and ranked positive signals therein by using the System Organ Class (SOC) and Preferred Term (PT) of the Adverse Drug Reaction Terminology Set, in order to identify new potential risk signals for the safe clinical use of this drug. In this study, for example, we mined the risk signals of Semaglutide and Liraglutide, which are commonly used in our hospital, and utilized the OpenVigil 2.1 data platform to “Semaglutide”, “Ozempic”, “Rybelsus”, “Wegovy”, “Liraglutide” and “Saxenda” as subject terms systematically searched the FAERS database to collect data from January 1, 2018 to September 30, 2023 and remove duplicate reports. Positive signal judgment criteria: one suspicious risk signal was generated when the number of ADE reports (a) ≥ 3, the lower limit of the 95% confidence interval (CI) of the ROR > 1 or PRR ≥ 2 and χ^2^ ≥ 4 [20].

### Study design and participants

This retrospective quasi-experimental study compared GLP-1RA prescription patterns before (April–June 2022) and after (April–June 2023) the implementation of information pharmacist-led audit rules integrating FAERS-based risk alerts for dosing errors and contraindications at The Affiliated Yan’an Hospital of Kunming Medical University. The intervention included standardized prescription reviews and risk alerts based on FAERS database.

All prescriptions for liraglutide or semaglutide during the study periods were extracted from the hospital’s electronic system. Duplicates and records with incomplete data (e.g., missing diagnosis, dosage, or duration) were excluded. Eligible participants were adults (≥ 18 years) with T2DM and complete prescription documentation (including diagnosis, dosage, treatment duration, and prescriber details). Exclusions comprised off-label uses (e.g., type 1 diabetes), contraindications (e.g., pregnancy, personal/family history of medullary thyroid carcinoma), or records with > 20% missing audit parameters (Tables 2 and 3).

### Evaluation of GLP-1RA prescription appropriateness

Following the development and implementation of GLP-1RA prescribing review rules by informatics pharmacists, we assessed prescription adherence and medication appropriateness for selected GLP-1RA agents used in our hospital. A comparative analysis was performed between the post-intervention period (April–June 2023) and the pre-intervention period (April–June 2022). 

To analyze the data, Statistical Package for the Social Sciences (SPSS) 22.0 statistical software was used for data calculation and processing. Since the data in this study are binary qualitative data, they are all described in the form of frequency and percentage, and expressed as (n, %), only Chi-square test is used for comparison. A p-value less than 0.05 was considered statistically significant.

### Summary of information sources

The information was obtained by retrieving and reviewing the latest authoritative large-scale reference books, manuals, and databases, including Martindale: The Complete Drug Reference (35th Edition, Chinese version), Chinese Pharmacopoeia Clinical Medication Guidelines (2020 Edition), New Guide to Pharmaceuticals (18th Edition), PubMed, MICROMEDEX, UpToDate, FAERS database, OpenVigil 2.1 data platform, CNKI, and Wanfang Medical Database.

## Results

### Summary of basic information of existing GLP-1RA in China

As of December 2023, there were 9 drugs in the GLP-1RA class approved for the treatment of T2DM in China, including Semaglutide injection, Liraglutide injection, Dulaglutide injection, Polyethylene glycol loxenatide injection, Insulin degludec and liraglutide injection, Exenatide injection, Benaglutide injection, Exenatide microsphees for injection, and Lixisenatide injection [21, 22]. The basic information of the drugs in this category is shown in Table 1, which contains information such as drug name, specification, medical insurance attributes, prescription restrictions, and whether it is a national base drug or not. The most commonly used GLP-1 RAs in our hospital are Semaglutide injection and Liraglutide injection.

**Table 1:** Summary of basic information on clinical application of GLP-1RA drugs

Common name	Specification	medical insurance level	Prescription restrictions	National basic drugs	National negotiated drugs	monotherapy	Reduce MACE in patients with T2DM and CVD
Exenatide injection	5ug(0.25 mg/ml, 1.2 ml); 10ug(0.25 mg/ml, 2.4mL)	Class B	√	×	√	×	×
Liraglutide injection	3 mL:18 mg	Class B	√	√	√	×	√
Benaglutide injection	21 ml:42 mg (420000U)	Class B	√	×	√	×	×
Exenatide microsphees for injection	2 mg	Class B	√	×	×	×	×
Lixisenatide injection	0.05 mg/ml,3 ml; 0.1 mg/ml,3mL	Class B	√	×	√	×	×
Dulaglutide injection	0.5 ml:0.75 mg; 0.5 ml:1.5 mg	Class B	√	×	√	√	*
Polyethylene glycol loxenatide injection	0.5mL:0.1 mg; 0.5mL:0.2 mg	Class B	√	×	√	√	×
Semaglutide injection	1.34 mg/ml,1.5 ml; 1.34 mg/ml,3 ml	Class B	√	×	√	×	√
Insulin degludec and liraglutide injection	3mL(Contains 300 units of Insulin Deglu and 10.8 mg of Liraglutide)	Class B	√	×	×	√	×

Note: “Medical insurance level ‘Class B’” refers to drugs partially reimbursed by China’s national medical insurance system, requiring patients to cover a proportion (typically 10–30%) of costs. “Prescription restrictions” indicates usage must meet specific criteria (e.g., approved indications, specialist-prescribed) for reimbursement eligibility. “National basic drugs” denotes medications listed in the National Essential Medicines Directory by National Health Commission, representing clinically essential, cost-effective medicines with guaranteed supply. “National negotiated drugs” are pharmaceuticals included in the reimbursement list through centralized price negotiations between NHSA and manufacturers, achieving significant price reductions

*CVD* Cardiovascular Disease, *MACE* Major Adverse Cardiovascular Events, *National Negotiated Drugs* Drugs whose payment standards are determined and included in the medical insurance catalog after price negotiations between the National Health Security Bureau and drug manufacturers

√: Yes, ×: No, *: cardiovascular benefit but no approved indication in China

### Creation and maintenance of GLP-1RA audit rules

The information pharmacists create GLP-1RA drug audit rules in combination with the actual situation of the hospital, including standardization of indications, dosage, medication for special populations, special pathological states, co-medication, interactions, and other audit judgment criteria, in order to warn the patients of prudent use, prohibited use, and ADEs. As shown in Table 2 and Table 3, taking liraglutide and semaglutide as examples, we focus on standardizing the contents requiring pharmacist audit encountered in the process of pre-prescription audit, paying attention to potential ADEs, and constructing corresponding audit specifications to reduce the pharmacist’s subjective factors affecting the judgment results. The rules are also applicable to the post-prescription review process, in which the audit rules are modified or added in time to ensure that the audit content is standardized and the warning level is reasonable.

**Table 2:** Liraglutide injection review rules

Audit Indicators	Audit Type	Audit Content	Audit Mode (pharmacist/system)	Warnings for exceeding rules
Indications	Diagnosis/ Patient Conditions	(1) Indicated for adult patients with T2DM who have poor glycemic control despite treatment with metformin and/or sulfonylureas based on dietary control and exercise. (2) Indicated to reduce the risk of major adverse cardiovascular events in adult patients with T2DM with concomitant cardiovascular disease	pharmacist	**not recommended**
Dosage	Adults	Subcutaneous injection. Starting dose 0.6 mg/day, after at least one week the dose should be increased to 1.2 mg or even 1.8 mg. Maximum daily dose is 1.8 mg.	system	**prohibited**
Special Populations	Elderly (> 65 years)	No need for dose adjustment based on age	system	concern
	Children	The safety and efficacy of this drug has not been established in children and adolescents under 18 years of age	system	caution
	Pregnant women	This drug should not be used during pregnancy	pharmacist	**prohibited**
	Lactating Women	Due to lack of relevant experience, this drug should not be used during lactation.	pharmacist	**prohibited**
Special Pathology	Renal impairment	Patients with mild, moderate or severe renal impairment do not require dose adjustment	system	concern
		Patients with end-stage renal disease: not recommended	pharmacist	**not recommended**
	Hepatic impairment	No dose adjustment is required in patients with mild or moderate hepatic impairment	system	concern
		Patients with severe hepatic impairment: not recommended	pharmacist	**not recommended**
	Type 1 Diabetes	Not to be used in patients with type 1 diabetes or for the treatment of diabetic ketoacidosis	system& pharmacist	**prohibited**
	Medullary Thyroid Cancer (MTC)	Not to be used in patients with a past or family history of MTC	system& pharmacist	**prohibited**
	Multiple endocrine neoplasia type 2 (MEN2)	Not to be used in patients with MEN2	system& pharmacist	**prohibited**
	Congestive heart failure	Not yet recommended for patients with congestive heart failure in cardiac class IV	pharmacist	**not recommended**
	Inflammatory bowel disease and diabetic gastroparesis	The treatment with this drug is accompanied by transient gastrointestinal adverse effects	pharmacist	**not recommended**
	Acute pancreatitis	Discontinue this drug if the disease is confirmed to have occurred	system	caution
Contraindications	Allergies	Hypersensitivity to the active ingredients of this drug or to any other excipients in this drug	system	**prohibited**
Combinations /Interactions	Sulfonylureas	The risk of hypoglycemia may be increased in patients receiving this product in combination with sulfonylureas. Consideration should be given to reducing the dose of sulfonylureas	system	concern
	Coumarins	More frequent INR testing is recommended when combining medications	system	concern
Adverse Reactions	From the drug insert	Reviewed and prompted by system rules	system& pharmacist	concern
	ADEs provided by FAERS	ADEs not listed in the drug insert but to be avoided in the context of the clinical situation	system& pharmacist	**concern&** **prohibited**

Note: Warnings for exceeding rules include prohibitions, non-recommendations, warnings, and concerns

**Table 3:** Semaglutide injection review rules

Audit Indicators	Audit Type	Audit Content	Audit Mode (pharmacist/system)	Warnings for exceeding rules
Indications	Diagnosis/ Patient Conditions	(1) Indicated for adult patients with T2DM who have poor glycemic control despite treatment with metformin and/or sulfonylureas based on dietary control and exercise. (2) Indicated to reduce the risk of major adverse cardiovascular events in adult patients with T2DM with concomitant cardiovascular disease	pharmacist	**not recommended**
Dosage	Adults	Subcutaneous injection. The starting dose is 0.25 mg weekly; after 4 weeks, the dose should be increased to 0.5 mg weekly. After at least 4 weeks of treatment with 0.5 mg weekly, the dose may be increased to 1 mg weekly. Doses greater than 1 mg per week are not recommended	system& pharmacist	**not recommended**
	Body weight	The heavier the body weight, the lower the exposure. 0.5 mg and 1 mg administered in the 40–198 kg body weight range provided adequate systemic exposure	pharmacist	**not recommended**
Special Populations	Pregnant women	Contraindicated in pregnancy. This drug should be discontinued at least 2 months before pregnancy is planned	pharmacist	**prohibited**
	Lactating Women	Due to lack of relevant experience, this drug should not be used during lactation	pharmacist	**prohibited**
	Children	The safety and efficacy of this drug has not been established in children and adolescents under 18 years of age	system	caution
	Elderly	No need for dose adjustment based on age	system	concern
Special Pathology	Renal impairment	No Dose Adjustment Needed in Patients with Mild, Moderate or Severe Renal Impairment	system	concern
		Not recommended for patients with end-stage renal disease	system& pharmacist	concern
	Hepatic impairment	No dose adjustment is required. Caution should be exercised when using this drug in patients with severe hepatic impairment	system	caution
	Acute pancreatitis	Discontinue this drug if the disease is confirmed to have occurred	system	caution
	Diabetic retinopathy	Caution should be exercised when adding this drug to patients receiving insulin therapy	system	caution
Contraindications	Type 1 Diabetes	Not to be used in patients with type 1 diabetes or for the treatment of diabetic ketoacidosis	pharmacist	**prohibited**
	MTC	Not to be used in patients with a past or family history of MTC	system& pharmacist	**prohibited**
	MEN2	Not to be used in MEN2 patients	system& pharmacist	**prohibited**
	Allergies	Hypersensitivity to the active ingredients of this drug or to any other excipients in this drug	system	**prohibited**
Combinations /Interactions	Oral medications	This drug should be used with caution in patients taking oral medications that require rapid gastrointestinal absorption	system	concern
Adverse Reactions	From the drug insert	Reviewed and prompted by system rules	system& pharmacist	concern
	ADEs provided by FAERS	ADEs not listed in the drug insert but to be avoided in the context of the clinical situation	system& pharmacist	**concern&** **prohibited**

Note: Warnings for exceeding rules include prohibitions, non-recommendations, warnings, and concerns

### Risk gating of ADEs in GLP-1RAs

In this study, the ADE risk signals of liraglutide and semaglutide were mined as an example. Database mining of FAERS yielded 48,389 semaglutide-related and 50,289 liraglutide-related adverse event reports. After screening and organizing, ADE data that satisfy both ROR and PRR were retrieved. In order of the number of reports (Table 4), the most common adverse events for liraglutide were nausea, hyperglycemia, vomiting, diarrhea, pancreatitis, decreased appetite, weight loss, headache, upper abdominal pain, and constipation. For semaglutide, the predominant events were nausea, vomiting, diarrhea, off-label use, decreased appetite, weight loss, constipation, headache, hyperglycemia, and fatigue. In order of signal strength (Table 5), “thyroid C-cell hyperplasia” and “drug dose titration not performed” topped the list of both, respectively. In addition, the signals of “blood calcitonin increased, pancreatic carcinoma metastatic, starvation ketoacidosis, lack of satiety, allodynia, hunger, and food craving” were not included in the drug insert. As shown in Table 6, based on the above 2 orderings, the SOCs of liraglutide risk signals were dominated by gastrointestinal system disorders, various types of investigations, systemic disorders and various reactions at the site of administration, and metabolic and nutritional disorders. The SOCs of the risk signals of semaglutide were mainly gastrointestinal disorders, various types of injuries, poisonings, and operational complications, systemic disorders, and various reactions at the site of administration. Through the above ADEs signals, the information pharmacist, when helping to audit the rationality of the use of this type of drugs, captures a full range of patient information to accurately prejudge potential ADEs, and prompts doctors and patients through the prescription pre-review system pop-up box prompts, verbal explanations, telephone notification, pharmacist checkups or medication monitoring, so as to minimize the risk of ADEs, increase patient satisfaction and acceptance, and improve the quality of medical care.

**Table 4:** Top 10 preferred terms by the number of reports

Sort	Liraglutide					Semaglutide				
	PT	Number of reports	ROR Lower limit of 95% CI	PRR	χ^2^	PT	Number of reports	ROR Lower limit of 95% CI	PRR	χ^2^
1	nausea	4431	46.87	5.78	17938.40	nausea	3264	6.18	5.44	12123.71
2	blood glucose increased^*^	1853	15.27	9.31	13610.31	vomiting	1982	6.04	5.74	7836.26
3	vomiting	1801	13.29	4.07	4220.45	diarrhoea	1547	3.44	3.40	2675.05
4	diarrhoea	1710	7.81	2.94	2231.06	off label use	1438	2.35	2.36	1166.41
5	pancreatitis	1429	6.07	25.15	31662.97	decreased appetite	1259	8.23	8.18	7901.60
6	Decreased appetite	1315	2.89	6.68	6321.97	weight decreased	1071	5.45	5.51	3966.22
7	weight decreased	1124	1.52	4.52	3091.01	constipation	882	5.91	6.06	3723.45
8	headache	1050	1.24	1.70	307.85	headache	868	1.71	1.79	312.61
9	abdominal pain upper	825	0.99	4.28	2072.49	blood glucose increased^*^	725	4.42	4.61	2051.64
10	constipation	765	0.87	4.10	1795.80	fatigue	666	1.08	1.16	15.95

Note：“PRR” refers to Proportional Reporting Ratio. “ROR” denotes Reporting Odds Ratio. “PT” means Preferred Term.

^*^indicates the ADE not mentioned in the instruction

**Table 5:** Top 10 preferred terms by signal strength

Sort	Liraglutide					Semaglutide				
	PT	Number of reports	ROR Lower limit of 95% CI	PRR	χ^2^	PT	Number of reports	ROR Lower limit of 95% CI	PRR	χ^2^
1	thyroid c-cell hyperplasia	3	127.96	765.77	916.52	drug dose titration not performed	155	134.23	158.70	19543.57
2	weight loss poor	262	75.22	84.82	18611.94	product communication issue	255	67.61	75.96	16901.42
3	blood calcitonin increased^*^	14	63.60	113.45	1276.68	weight loss poor	188	64.59	74.39	12221.39
4	medullary thyroid cancer	16	42.85	72.28	985.28	medullary thyroid cancer	18	64.43	105.93	1609.89
5	lack of satiety^*^	12	36.50	66.59	685.80	starvation ketoacidosis^*^	7	61.31	138.57	788.717
6	product contamination with body fluid	14	33.18	57.64	700.14	allodynia^*^	34	47.79	67.92	2030.745
7	eructation	503	30.14	32.32	14366.33	obstructive pancreatitis	22	47.24	73.32	1411.056
8	pancreatic carcinoma metastatic^*^	83	28.45	35.43	2596.98	lack of satiety^*^	10	36.22	69.49	610.1768
9	pancreatitis	1429	25.33	25.15	31662.97	injection site discharge	73	34.64	43.75	2858.246
10	thyroid adenoma	8	23.02	47.49	333.10	eructation	394	29.48	31.95	11270.84

Note: “PRR” refers to Proportional Reporting Ratio. “ROR” denotes Reporting Odds Ratio. “PT” means Preferred Term.

^*^indicates the ADE not mentioned in the instruction

**Table 6:** Composition ratio of signals and reported numbers in SOC

Cumulative SOC	**Liraglutide**			**Liraglutide**		
	Number of signals	PT		Number of signals	PT	
		Number of signals	Component ratio/%		Number of reports	Component ratio/%
Gastrointestinal System Diseases	48	15,331	46.87	69	12,834	38.41
Various examinations	24	4994	15.27	29	3134	9.38
Systemic diseases and reactions at the site of administration	35	4347	13.29	26	3653	10.93
Metabolic and nutritional diseases	19	2556	7.81	28	2879	8.62
Various neurological disorders	8	1986	6.07	19	2131	6.38
Benign, malignant and tumors of unknown nature (including cystic and polypoid)	31	944	2.89	12	208	0.62
Product problems	6	497	1.52	17	827	2.48
Hepatobiliary system diseases	12	406	1.24	14	449	1.34
Diseases of the skin and subcutaneous tissue	7	325	0.99	11	565	1.69
Musculoskeletal and connective tissue diseases	2	286	0.87	3	56	0.17
Injuries, poisoning and operational complications of all kinds	8	246	0.75	36	4125	12.35
Diseases of the heart	1	148	0.45	4	149	0.45
Diseases of the respiratory system, chest and mediastinum	2	137	0.42	5	182	0.54
Diseases of the kidney and urinary system	6	110	0.34	6	318	0.95
Diseases of the endocrine system	6	108	0.33	4	71	0.21
Infectious and invasive diseases	5	96	0.29	9	372	1.11
Surgery and medical procedures	9	93	0.28	15	236	0.71
Social Environment	2	43	0.13	2	73	0.22
Diseases of the Reproductive System and Breasts	4	41	0.13	6	77	0.23
Diseases of the eye organ	1	14	0.04	17	820	2.45
Psychiatric disorders	−	−	−	8	234	0.70
Diseases of the ear and labyrinth	−	−	−	2	11	0.03
Pregnancy, puerperium and perinatal conditions	−	−	−	1	4	0.01

Note: “PT” means Preferred Term

### Evaluation of GLP-1RA medication rationality

Using liraglutide and semaglutide (the most prescribed GLP-1RAs in our hospital) as examples, Tables 7, 8 and 9, 10 show that implementing standardized audit rules significantly reduced irrational prescriptions in 2023 (April–June) versus 2022 (*p* < 0.001). Specifically, Tables 7 and 8 demonstrate the improvement in liraglutide prescription rationality, while Tables 9 and 10 reveal a similar trend for semaglutide, with a notable reduction in unreasonable prescriptions after the implementation of audit rules. Prescription appropriateness was determined based on the manufacturer’s prescribing information and information pharmacist-established review criteria.

**Table 7:** Comparison of the rationality of liraglutide injection prescriptions before and after the implementation of audit rules in different months of the same year

Year	Month	Number of unreasonable prescriptions	Number of reasonable prescriptions	X^2^	*P*
2022	April	12 (35.3)	22 (64.7)	1.394	0.498
	May	12 (33.3)	24 (66.7)		
	June	10 (23.8)	32 (76.2)		
2023	April	16 (10.9)a	131 (89.1)	11.990	0.002
	May	7 (3.8)b	177 (96.2)		
	June	4 (2.5)b	156 (97.5)		

Note: The results of the group labeled “a” were compared with those of the group labeled” b “, and the difference was statistically significant

**Table 8:** Comparison of the rationality of liraglutide injection prescriptions before and after the implementation of audit rules in the same month

Month	Year	Number of unreasonable prescriptions	Number of reasonable prescriptions	X^2^	*P*
April	2022	12 (35.3)	22 (64.7)	12.582	< 0.001
	2023	16 (10.9)	131 (89.1)		
May	2022	12 (33.3)	24 (66.7)	33.273	< 0.001
	2023	7 (3.8)	177 (96.2)		
June	2022	10 (23.8)	32 (76.2)	23.420	< 0.001
	2023	4 (2.5)	156 (97.5)		

**Table 9:** Comparison of the rationality of semaglutide injection prescriptions before and after the implementation of audit rules in different months of the same year

Year	Month	Number of unreasonable prescriptions	Number of reasonable prescriptions	X^2^	*P*
2022	April	49 (25.7)	142 (74.3)	3.132	0.209
	May	56 (32.2)	118 (67.8)		
	June	61 (33.5)	121 (66.5)		
2023	April	1 (0.8)	129 (99.2)	0.494	0.781
	May	2 (0.9)	216 (99.1)		
	June	3 (1.5)	197 (98.5)		

**Table 10:** Comparison of the rationality of semaglutide injection prescriptions before and after the implementation of audit rules in the same month

Month	Year	Number of unreasonable prescriptions	Number of reasonable prescriptions	X^2^	*P*
April	2022	49 (25.7)	142 (74.3)	36.427	< 0.001
	2023	1 (0.8)	129 (99.2)		
May	2022	56 (32.2)	118 (67.8)	75.037	< 0.001
	2023	2 (0.9)	216 (99.1)		
June	2022	61 (33.5)	121 (66.5)	70.033	< 0.001
	2023	3 (1.5)	197 (98.5)		

## Discussion

GLP-1RA is a new type of hypoglycemic drug used in the treatment of T2DM, which is widely used in clinical practice at home and abroad due to its reliable and safe efficacy [23–25]. Notably, GLP-1RA differs from traditional antidiabetic drugs due to the broad distribution of GLP-1R across multiple organs, including the pancreas, central nervous system, cardiovascular system, liver, adipose tissue, and skeletal muscle. This enables GLP-1RA to exert multisystem effects beyond glycemic control, providing additional benefits such as weight reduction, blood pressure regulation, lipid profile improvement, and hepatorenal protection in T2DM management [26–28]. At present, multiple GLP-1RAs had been approved in China, significantly expanding treatment options for T2DM.

Current evidence indicates substantial knowledge gaps concerning GLP-1RAs among Chinese physicians. A recent Multicenter survey revealed that 40.2% of participating clinicians had never prescribed this drug class, with key barriers including high treatment costs, suboptimal patient adherence, safety concerns, and insufficient therapeutic knowledge [29]. Given that GLP-1RA therapy requires careful management of administration routes, dosage regimens, drug interactions, and adverse effects, comprehensive pharmaceutical oversight - including prospective prescription review, retrospective prescription analysis, and ongoing therapeutic monitoring - is clinically essential. As an important pharmacy personnel in drug information acquisition, organization, storage and delivery, hospital information pharmacists need to provide reliable decision support data for hospital rational medication management, clinical decision-making and patients’ medication counseling [30–33]. Therefore, the information pharmacist team of our hospital pays close attention to the new drug dynamics of GLP-1RA drugs, the information of post-marketing re-evaluation of drugs, pharmacological properties, clinical application, occurrence of adverse reactions, etc. Based on GLP-1RA prescribing guidelines and our hospital’s needs, we developed audit rules to standardize dosing (dosage form, frequency, and subcutaneous-only administration), enforce contraindication checks, and prohibit mixing with incompatible drugs. In addition, the information pharmacists mined and analyzed the ADEs data of GLP-1RA in the FAERS database through ROR and PRR [34, 35], with a view to discovering new potential risk signals and providing effective references for the safe clinical use of the GLP-1RA class of drugs. Regarding the handling of possible medication use beyond the limited scope of the drug specification, clinicians are required to fill out and submit the “Application form for filing of off-label drug use” developed with the assistance of the information pharmacist, as well as the corresponding evidence-based medical evidence [36, 37], which can only be implemented after consideration and approval by the hospital. On this basis, the information pharmacists can modify or add new drug rules according to the content of the application in order to collaborate with the clinical pharmacist to do a good job of pharmacy monitoring and patient medication education.

Regarding the formation and development of hospital information pharmacists, more and more hospitals at home and abroad have already emphasized and developed information pharmacists, and all of them have presented successful pharmacy intervention models, thus effectively promoting the rational use of medication in the clinic and avoiding medication risks. A U.S. multi-hospital health informatics study demonstrated the essential role of information pharmacists, who collaborate with information technology specialists to develop data-driven strategies for optimizing medication safety through advanced information systems [38]. The University of Nottingham School of Medicine in the United Kingdom has successfully reduced the rate of hazardous and erroneous prescriptions, and effectively controlled and reduced the risk indicators of adverse reactions, such as gastrointestinal bleeding, through the large-scale promotion and implementation of technological interventions by information pharmacists in clinical practice [39]. Therefore, hospital-based information pharmacists are worth promoting and developing. At present, our hospital is also expanding the number of information pharmacists and fully training and improving their overall academic level and practical skills to make up for the existing shortcomings such as fewer members, varying academic qualifications and work experience, and the inability to adequately guide and standardize the rational use of all key monitored drugs.

Our study was mainly based on the hospital’s further strengthening of its medical information technology platform, during which the medical team was basically fixed without any quality improvement program, which eliminated the bias in results due to personnel differences and quality adjustments, and provided reliability and practicality. This project is also the creation and use of the prescription pre-review system and the promotion of the prescription post-review work, which is in line with the actual situation and needs of our hospital’s clinical medication, and is instructive and implementable. In addition, this is the first practical work of our team, which has achieved preliminary results with validity and novelty. We will also further expand the new model of hospital pharmacy service, carry out the information pharmacist intervention and control of key drugs, and promote the use of the information pharmacist in the hospital and outside the hospital.

Despite the promising findings, this study has several limitations. First, the sample size was relatively small, which may limit the generalizability of the results. Second, only a limited number of GLP-1RA drugs were included in the analysis, and the study did not cover all available agents in this class, potentially affecting the comprehensiveness of the prescription review rules. Third, the study was conducted in a single tertiary hospital in China, which may not fully represent the prescribing practices and patient populations in other regions or healthcare settings. Additionally, the study only examined data from a specific time period, and longer-term observations may be necessary to assess the sustained impact of the implemented rules. Future multicenter studies with larger sample sizes, broader drug coverage, and extended follow-up periods are warranted to validate and refine these findings.

## Conclusions

This study demonstrates the pivotal role of information pharmacists in developing GLP-1RA prescription review rules, enhancing medication safety through standardized protocols, contraindication alerts, and evidence-based off-label use management. While limited by its single-center design and short-term observations, our findings provide a practical framework for optimizing GLP-1RA use. Future multicenter studies with extended follow-ups are needed to validate and expand this model. The continued development of information pharmacists will be crucial for advancing intelligent pharmacy services and ensuring patient-centered medication safety.

## Data Availability

All data generated or analysed during this study are included in this published article.

## Abbreviations

GLP-1RA: Glucagon-like peptide-1 receptor agonist
T2DM: Type 2 Diabetes Mellitus
ADEs: Adverse Drug Events
FAERS: FDA Adverse Event Reporting System
ROR: Reporting Odds Ratio
PRR: Proportional Reporting Ratio
SOC: System Organ Class
PT: Preferred Term
CI: Confidence Interval
